# Supplementary material for: Economics of vaccination against diarrhoea and respiratory diseases in French cow calves’ systems: A modelling approach
Source: PLoS One. 2025 Aug 6;20(8):e0329325. doi: 10.1371/journal.pone.0329325 (PMC12327687; doi:10.1371/journal.pone.0329325)

**Supplementary Material – Economics of vaccination against diarrhoea and respiratory diseases in French cow calves’ systems: a modelling approach**

Ferchiou Ahmed^1^*, Giacomini Anthony^1^, Herman Nicolas^2^, Lhermie Guillaume^1,3^, Raboisson Didier^1^

^1^ : UMR ASTRE, Université de Toulouse, CIRAD, INRAE, ENVT, Toulouse, France

^2^ : Clinique Vétérinaire Des Mazets, Les Mazets, Riom-ès-Montagnes 15400, France

^3^ : Faculty of Veterinary Medicine, University of Calgary, Calgary T2N 4Z6, AB, Canada

* : corresponding author

[S1 Table 1: List of calculated indicators 2](#_Toc203144557)

[S1 Table 2: Treatment protocol for cows and calves 5](#_Toc203144558)

[S1 Table 3: Characteristics of medicines used for cows and calves’ diseases 6](#_Toc203144559)

[S1 Table 4: Other output results of the bioeconomic model: mortality rates 7](#_Toc203144560)

[S1 Table 5: Other output results of the bioeconomic model: other economic outputs of the model 8](#_Toc203144561)

[S1 Figure 1: Other output results of the bioeconomic model: average animal presence per year and per scenario 9](#_Toc203144562)

[S1 Figure 2: Other output results of the bioeconomic model: Graphical representation of the reduction in the prevalence of respiratory diseases and diarrhea. 10](#_Toc203144563)

[S1 Figure 3: Other output results of the bioeconomic model: Graphical representation of the reduction in calf’s mortality rates 11](#_Toc203144564)

[S1 Figure 4: Average simulated annual cases of Diarrhea and Respiratory Diseases across scenarios with and without vaccination 12](#_Toc203144565)

# S1 Table 1: List of calculated indicators

| **Indicators** | **Definition** (unit) | **Value** |
| --- | --- | --- |
| **Herdsize** | Number of cows (cow year count) | 99.67  (1.61) |
| **Hsize_Calves_1mo_** | Number of males and females present at 1 month of age | 106.11  (1.51) |
| **Hsize_Calves_6mo_** | Number of males and females present at 6 months of age | 101.17  (1.86) |
| **Hsize_Calves_10mo_** | Number of males and females present at 10 months of age | 78.22  (1.24) |
| **MorRate** | Cow mortality rate (Number of dead cows / Herdsize) | 2.01  (0.14) |
| **ReplacementRate** | Replacement rate (Number of first calvings x 100 / Herdsize) | 26.03  (1.35) |
| **CullingRate** | Culling rate (Number of culled cows excluding mortality x 100 / Herdsize) | 24.26  (2.06) |
| **WeightCows** | Total carcass weight of culled cows (kg) | 9245.72  (29.54) |
| **CalvInterval** | Average calving-to-calving interval (d) | 396.84  (0.98) |
| **AgeCalv1** | Average age of first calvings (wks) | 161.66  (0.77) |
| **Nbirth_Male_** | Number of male births | 55.18  (0.92) |
| **Nbirth_Female_** | Number of female births | 55.21  (0.91) |
| **FemSold** | Number of heifers sold | ·· |
| **MalSold** | Number of male calves sold | ·· |
| **FemWeight** | Average weight of females sold (kg) | 385.77  (0.51) |
| **MalWeight** | Average weight of males sold (kg) | 308.97  (1.55) |
| **AgeFem** | Average age of females sold (wks) | ·· |
| **AgeMal** | Average age of males sold (wks) | ·· |
| **MorRate_M_** | Male mortality rate (Number of stillbirths + deaths / number of calvings) | ·· |
| **MorRate_F_** | Female mortality rate (Number of stillbirths + deaths / number of calvings) | ·· |
| **MorRate_M0-2j_** | Male mortality rate 0–2 days (number of stillbirths x 100 / number of calvings) | 1.53  (0.16) |
| **MorRate_F0-2j_** | Female mortality rate 0–2 days (number of stillbirths x 100 / number of calvings) | 1.55  (0.18) |
| **MorRate_M2j-1m_** | Male mortality rate 2 days–1 month excluding stillbirths  (number of males dead under one month / number of calvings) | ·· |
| **MorRate_F2j-1m_** | Female mortality rate 2 days–1 month excluding stillbirths  (number of males dead under one month / number of calvings) | ·· |
| **MorRate_M1m-6m_** | Male mortality rate 1–6 months including stillbirths  (number of males dead under 6 months / number of calvings) | ·· |
| **MorRate_F1m-6m_** | Female mortality rate 1–6 months including stillbirths  (number of males dead under 6 months / number of calvings) | ·· |
| **NbVac_Dia_** | Number of vaccines administered against diarrhoea | 110.47  (0.14) |
| **NbVac_BRD_** | Number of vaccines administered against respiratory diseases | 311.32  (1.27) |
| **NbCows_Vac_Dia_** | Number of cows vaccinated against diarrhoea | 110.47  (0.14) |
| **NbCalves_Vac_BRD_** | Number of individuals who received a vaccine dose against respiratory diseases | 153.16  (0.45) |
| **NbCases_Dia_** | Numbers of diarrhoea cases | ·· |
| **NbCases_BRD_** | Number of respiratory disease cases | ·· |
| **NbIndiv_Dia_** | Number of animals with diarrhoea | ·· |
| **NbIndiv_BRD_** | Number of animals with respiratory diseases | ·· |
| **Inci_Dia_** | Diarrhoea incidence (NbIndiv_Dia_ / Number of births) | ·· |
| **Inci_BRD_** | BRD incidence (NbIndiv_BRD_ / Number of births) | ·· |
| **Prev_Dia_** | Diarrhoea prevalence (NbCases_Dia_ / Number of births) | ·· |
| **Prev_BRD_** | BRD prevalence (NbCases_BRD_ / Number of births) | ·· |
| **ALEA_calves_1m_** | ALEA 0–1 (mo)  (sum of live weights of animals under one month treated with antibiotics / sum of treatable live weights of animals under one month) | ·· |
| **ALEA_calves_6m_** | ALEA 0–6 months  (sum of live weights of animals under six months treated with antibiotics / sum of treatable live weights of animals under six months) | ·· |
| **ALEA_allcalves** | ALEA for all calves  (sum of live weights of all calves treated with antibiotics / sum of treatable live weights of all calves) | ·· |
| **ALEA_critical** | ALEA critical antibiotics  (sum of live weights of animals treated with critical antibiotics / sum of treatable live weights of animals) | ·· |
| **nb_atb_calves_** | Number of antibiotic treatments administered to animals before calving | ·· |
| **nb_atb_Dia_** | Number of antibiotic treatments administered for diarrhoea cases | ·· |
| **nb_atb_Omph_** | Number of antibiotic treatments administered for omphalitis cases | ·· |
| **nb_atb_Sept_** | Number of antibiotic treatments administered for septicaemia cases | ·· |
| **nb_atb_BRD_** | Number of antibiotic treatments administered for respiratory disease cases | ·· |
| **InterVet1** | Number of level 1 veterinary interventions (20 min) | ·· |
| **InterVet2** | Number of level 2 veterinary interventions (30 min) | ·· |
| **InterVet3** | Number of level 3 veterinary interventions (over 45 min) | ·· |
| **Labor** | Farmer’s labour time for treatment, restraint for veterinary intervention, and vaccination of animals (unit) | ·· |
| **FemaleSold** | Revenue from sold females (€) | ·· |
| **MaleSold** | Revenue from sold males (€) | ·· |
| **CullSold** | Revenue from culled cows (€/year) | ·· |
| **Cost_Med_** | Total cost of medicine purchases (€/ year) | ·· |
| **Cost_Vac_** | Total cost of vaccine purchases (€/ year) | ·· |
| **Cost_Vet_** | Total cost of veterinary interventions (€/ year) | ·· |
| **FeedCost_Total_** | Total feed expenditure (€/ year) | ·· |
| **FeedCost_Male_** | Average feed expenditure per male sold (€/ head) | ·· |
| **FeedCost_Female_** | Average feed expenditure per female sold (€/ head) | ·· |

# S1 Table 2: Treatment protocol for cows and calves

|  | ***P_Trt_* (Actor)** | **Option # (P_Prot_)** | **Treatment** | **Time for farmer** (min) |
| --- | --- | --- | --- | --- |
| **Rpl** | 75% (F) | 1 (33%) | delivrance_manuelle + clamoxyl_oblets | 25 |
|  |  | 2 (33%) | Shotapen | 10 |
|  |  | 3 (33%) | delivrance_manuelle + Shotapen | 30 |
|  | 25% (V) | 1 (50%) | delivrance_manuelle | 15 |
|  |  | 2 (50%) | delivrance_manuelle + Shotapen + clamoxyl_oblets | 15 |
| **Met** | 25% (F) | 1 (50%) | Shotapen + Tolfine | 15 |
|  |  | 2 (50%) | Histabiosone | 30 |
|  | 75% (V) | 1 (100%) | duphamox_LA + Tolfine | 30 |
| **Pvd** | 75% (F) | 1 (50%) | no_trt | 0 |
|  |  | 2 (50%) | Estrumate | 10 |
|  | 25% (V) | 1 (100%) | Estrumate + Metricure | ·· |
| **BRD** | 90% (F) | 1 (50%) | Nuflor + Tolfine | 15 |
|  |  | 2 (50%) | Draxxin100 + Tolfine | 15 |
|  |  | 1* (50%) | Nuflor + Tolfine + Dexadreson | 15 |
|  |  | 2* (50%) | Draxxin100 + | 15 |
|  | 10% (V) | 1 (50%) | Nuflor + Tolfine | 15 |
|  |  | 2 (50%) | Marbocyl10 + Dexadreson | 15 |
|  |  | 1 (50%) | Nuflor + Tolfine | 15 |
|  |  | 2 (50%) | Marbocyl10 + Dexadreson | 15 |
| **Omph** | 100% | 1 (100%) | Histabiosone | 15 |
| **Sep** | 25% (F) | 1 (75%) | Potencil + Tolfine + Dexadreson | 30 |
|  |  | 2 (25%) | Multibio | 10 |
|  | 75% (V) | 1 (33%) | Marbocyl10 + Speciale2411 + Tolfine | 45 |
|  |  | 2 (33%) | G4 + Speciale2411 + Tolfine | 45 |
|  |  | 3 (33%) | Amphoprim + Speciale2411 + Tolfine | 45 |
| **Dia** | 90% (F) | 1 (50%) | Gabbrovet + Rehydion | 20 |
|  |  | 2 (25%) | Potencil + Rehydion | 20 |
|  |  | 3 (25%) | Gabbrovet + Multibio + Rehydion | 20 |
|  | 10% (V) | 1 (25%) | Marbocyl10 + Speciale2411 + Tolfine | 45 |
|  |  | 2 (25%) | G4 + Speciale2411 + Tolfine | 45 |
|  |  | 3 (25%) | Amphoprim + Speciale2411 + Tolfine | 45 |
|  |  | 4 (25%) | Speciale2411 + Tolfine | 45 |

# S1 Table 3: Characteristics of medicines used for cows and calves’ diseases

| **Commercial name** | **Molecule** | **Nature** | **Route** | **NA** | **Duration**  (days) | **Milk WT** (days) | **Meat WT** (days) |
| --- | --- | --- | --- | --- | --- | --- | --- |
| **Duphamox_LA** | Amoxicillin | AM | IM | 2 | 2 | 2.5 | 21 |
| **Dexadreson** | Dexamethasone | AIS | IM | 1 | 1 | 3 | 8 |
| **Nuflor** | Florfenicol | AM | SC | 1 | 1 | *prohibited* | 64 |
| **Draxxin100** | Tulathromycin | AM | SC | 1 | 1 | *prohibited* | 22 |
| **Tolfine** | Tolfenamic acid | AINS | IM | 1 | 2 | 0 | 12 |
| **Shotapen** | Benzylpenicillin, Dihydrostreptomycin | AM | IM | 2 | 3 | 5 | 64 |
| **Potencil** | Amoxicillin, Colistin | AM | IM | 2 | 3 | *for calves* | 10 |
| **Amphoprim** | Sulfadimidine, Trimethoprim | AM | IM | 3 | 1 | 2 | 5 |
| **Estrumate** | Cloprostenol | H | IM | 1 | 1 | 0 | 1 |
| **Speciale2411** | Sodium bicarbonate, Glucose, Saccharose | ME | IV | 3 | 1 | *for calves* | 0 |
| **Rehydion** | Sodium, Potassium, Glucose | REH | OR | 2 | 0.5 | 0 | 0 |
| **Metricure** | Cefapirin | AM | IVG | 1 | 1 | 0 | 2 |
| **Clamoxyl_Oblets** | Amoxicillin | AM | IU | 1 | 1 | 0 | 1 |
| **Histabiosone** | Benzylpenicillin, Dihydrostreptomycin, Chlorphenamine, Dexamethasone | AM | IM | 4 | 3 to 5 | 4.5 | 30 |
| **Marbocyl10** | Marbofloxacin | AM | IV | 1 | 3 to 5 | 1.5 | 6 |
| **G4** | Gentamicin | AM | IV | 1 | 3 | *prohibited* | 214 |
| **gabbrovet** | Paromomycin | AM | OR | 1 | 3 to 5 | 0 | 20 |
| **Multibio** | Ampicillin, Colistin, Dexamethasone | AM | IM | 3 | 3 | 3 | 21 |

Medicines are supposed to be used at their approved dose, NA denotes the number of applications per animal, Milk WT milk withdrawal time and Meat WT: meat withdrawal time. For treatments routes abbreviations, IM: intramuscular, IMA: intramammary, OR: oral, IU: intrauterine, IVG: intravaginal, IV: intravenous, SC: subcutaneous. For treatment nature abbreviations, AM: Antimicrobial, AIS: steroidal anti-inflammatory drug, AINS: Non-steroidal anti-inflammatory drug H: Hormones, ME: infusions for metabolic troubles, REH: rehydrating.

# S1 Table 4: Other output results of the bioeconomic model: mortality rates

| **Scenarios** | | **MorRate_M2j-1m_** | | **MorRate_F2j-1m_** | | **MorRate_M6m_** | | **MorRate_F6m_** | | **MorRate_M_** | | **MorRate_F_** | |
| --- | --- | --- | --- | --- | --- | --- | --- | --- | --- | --- | --- | --- | --- |
| **Risk** | **Vacc** | **Value** | **∆% Vacc00** | **Value** | **∆% Vacc00** | **Value** | **∆% Vacc00** | **Value** | **∆% Vacc00** | **Value** | **∆% Vacc00** | **Value** | **∆% Vacc00** |
| **Basic** | **00** | 6.46 | ·· | 5.28 | ·· | 9.97 | ·· | 8.56 | ·· | 11.96 | ·· | 10.84 | ·· |
|  | **0R** | 6.23 | -3.48% | 5.06 | -4.25% | 9.83 | -1.45% | 8.36 | -2.35% | 11.79 | -1.41% | 10.40 | -4.12% |
|  | **D0** | 5.36 | -16.94% | 4.45 | -15.71% | 9.04 | -9.32% | 7.93 | -7.35% | 11.02 | -7.83% | 10.10 | -6.82% |
|  | **DR** | 4.96 | -23.24% | 4.30 | -18.60% | 8.48 | -14.92% | 7.71 | -9.99% | 10.50 | -12.25% | 9.96 | -8.13% |
| **0.5** | **00** | 5.78 | ·· | 4.90 | ·· | 9.20 | ·· | 8.33 | ·· | 11.21 | ·· | 10.60 | ·· |
|  | **0R** | 5.73 | -0.79% | 4.64 | -5.24% | 9.13 | -0.78% | 7.95 | -4.56% | 11.13 | -0.79% | 10.12 | -4.57% |
|  | **D0** | 5.33 | -7.74% | 4.20 | -14.22% | 8.75 | -4.88% | 7.56 | -9.31% | 10.79 | -3.80% | 9.71 | -8.43% |
|  | **DR** | 4.98 | -13.84% | 4.13 | -15.58% | 8.45 | -8.20% | 7.42 | -11.01% | 10.57 | -5.79% | 9.59 | -9.54% |
| **1.5** | **00** | 6.95 | ·· | 5.65 | ·· | 10.66 | ·· | 9.09 | ·· | 12.59 | ·· | 11.26 | ·· |
|  | **0R** | 6.63 | -4.60% | 5.46 | -3.28% | 10.18 | -4.48% | 8.95 | -1.56% | 12.24 | -2.80% | 11.06 | -1.81% |
|  | **D0** | 5.35 | -22.93% | 4.36 | -22.88% | 9.00 | -15.58% | 7.79 | -14.30% | 11.12 | -11.64% | 10.05 | -10.72% |
|  | **DR** | 5.20 | -25.12% | 4.17 | -26.11% | 8.53 | -19.98% | 7.65 | -15.85% | 10.43 | -17.12% | 9.92 | -11.91% |

# S1 Table 5: Other output results of the bioeconomic model: other economic outputs of the model

| **Scenarios** | | **FeedCost_Total_** (€) | | **FeedCost_Male_** (€) | | **FeedCost_Female_** (€) | | **Cost_Vet_** (€) | | **Cost_Med_** (€) | | **Cost_Vacc_** (€) |
| --- | --- | --- | --- | --- | --- | --- | --- | --- | --- | --- | --- | --- |
| **Risk** | **Vacc** | **Value** | **∆% Vacc00** | **Value** | **∆% Vacc00** | **Value** | **∆% Vacc00** | **Value** | **∆% Vacc00** | **Value** | **∆% Vacc00** | **Value** |
| **Basic** | **00** | 44781.92 | ·· | 190.81 | ·· | 154.36 | ·· | 992.38 | ·· | 2898.48 | ·· | 0 |
|  | **0R** | 44563.28 | -0.49% | 185.97 | -2.53% | 149.36 | -3.24% | 869.13 | -12.42% | 2206.63 | -23.87% | 1862 |
|  | **D0** | 44836.79 | 0.12% | 189.66 | -0.60% | 152.93 | -0.93% | 949.12 | -4.36% | 2773.79 | -4.30% | 1099 |
|  | **DR** | 44652.25 | -0.29% | 184.98 | -3.05% | 148.41 | -3.86% | 802.12 | -19.17% | 2074.77 | -28.42% | 2974 |
| **0.5** | **00** | 44825.30 | ·· | 187.44 | ·· | 151.17 | ·· | 764.53 | ·· | 1918.09 | ·· | 0 |
|  | **0R** | 44593.48 | -0.52% | 185.53 | -1.02% | 149.06 | -1.40% | 709.82 | -7.16% | 1551.91 | -19.09% | 1868 |
|  | **D0** | 44921.79 | 0.22% | 186.91 | -0.28% | 150.53 | -0.42% | 732.80 | -4.15% | 1825.62 | -4.82% | 1104 |
|  | **DR** | 44746.54 | -0.18% | 184.92 | -1.34% | 148.57 | -1.72% | 660.38 | -13.62% | 1472.57 | -23.23% | 2981 |
| **1.5** | **00** | 44894.22 | ·· | 195.07 | ·· | 157.48 | ·· | 1233.76 | ·· | 3893.63 | ·· | 0 |
|  | **0R** | 44506.43 | -0.86% | 186.58 | -4.35% | 150.05 | -4.72% | 1043.34 | -15.43% | 2886.21 | -25.87% | 1856 |
|  | **D0** | 44952.68 | 0.13% | 193.47 | -0.82% | 155.36 | -1.34% | 1145.67 | -7.14% | 3707.77 | -4.77% | 1100 |
|  | **DR** | 44637.56 | -0.57% | 184.87 | -5.23% | 148.34 | -5.80% | 961.16 | -22.10% | 2680.69 | -31.15% | 2979.35 |

# S1 Figure 1: Other output results of the bioeconomic model: average animal presence per year and per scenario


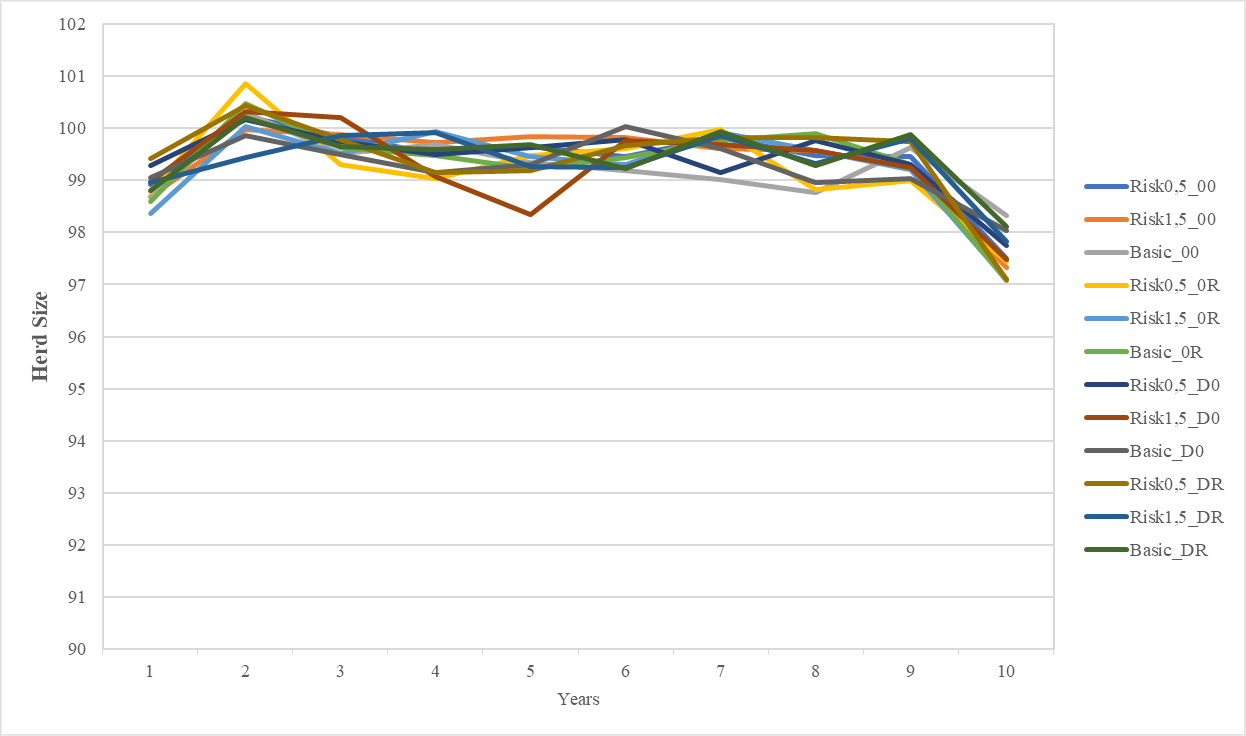


# S1 Figure 2: Other output results of the bioeconomic model: Graphical representation of the reduction in the prevalence of respiratory diseases and diarrhea.


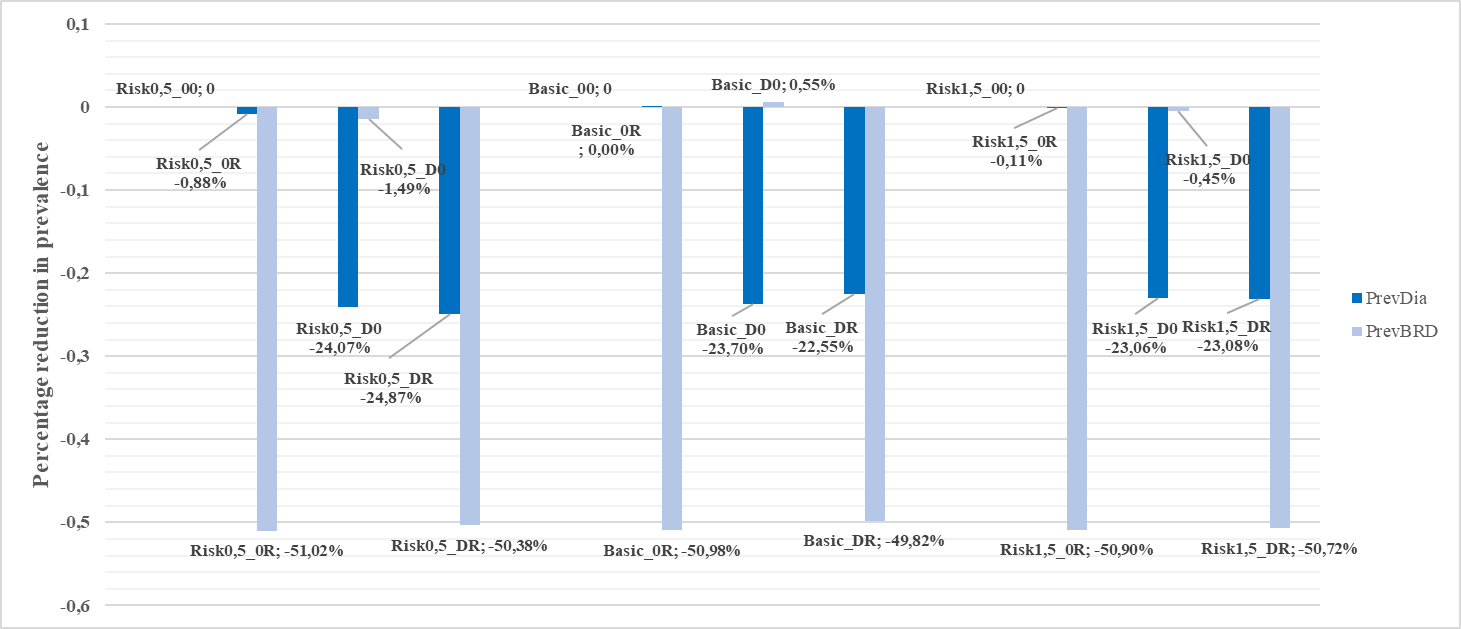


# S1 Figure 3: Other output results of the bioeconomic model: Graphical representation of the reduction in calf’s mortality rates


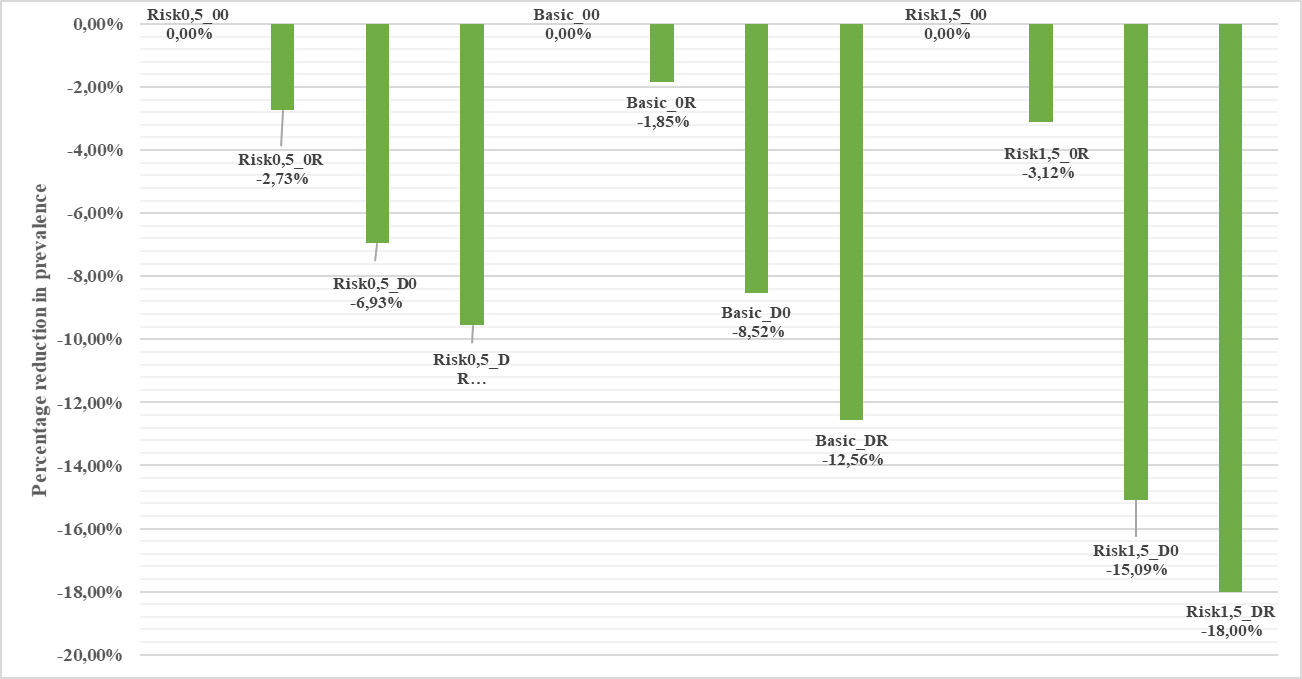


# S1 Figure 4: Average simulated annual cases of Diarrhea and Respiratory Diseases across scenarios with and without vaccination


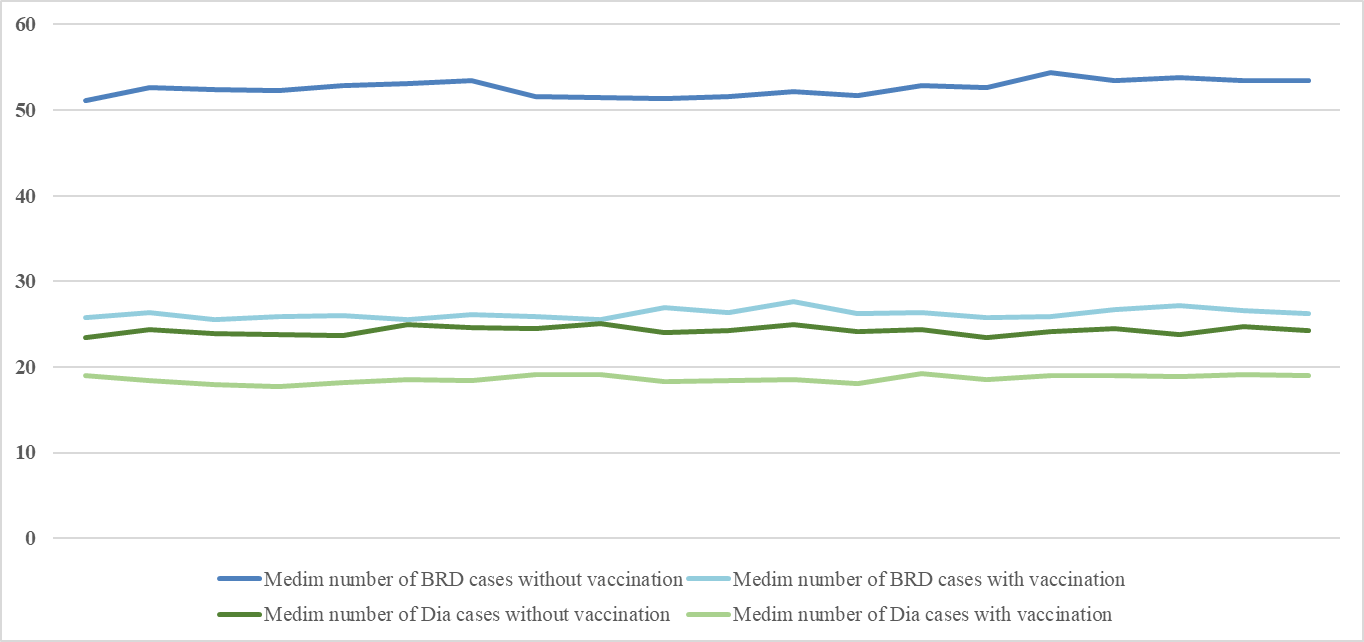

Supplement: S1 File — S2 Table: Treatment protocol for cows and calves. S3 Table: Characteristics of medicines used for cows and calves’ diseases. S4 Table: Other output results of the bioeconomic model: mortality rates. S5 Table: Other output results of the bioeconomic model: other economic outputs of the model. S1 Fig: Other output results of the bioeconomic model: average animal presence per year and per scenario. S2 Fig: Other output results of the bioeconomic model: Graphical representation of the reduction in the prevalence of respiratory diseases and diarrhea. S3 Fig: Other output results of the bioeconomic model: Graphical representation of the reduction in calf’s mortality rates. S4 Fig: Average simulated annual cases of Diarrhea and Respiratory Diseases across scenarios with and without vaccination. (DOCX) [file pone.0329325.s001.docx]
