# Supplementary material for: Economics of vaccination against diarrhoea and respiratory diseases in French cow calves’ systems: A modelling approach
Source: PLoS One. 2025 Aug 6;20(8):e0329325. doi: 10.1371/journal.pone.0329325 (PMC12327687; doi:10.1371/journal.pone.0329325)
Supplement: Attachment — Submitted filename: Response to reviewers 2025.04.15.docx [file pone.0329325.s004.docx]

**Dear Reviewers and Editor,**

**Below are our responses to the comments and suggestions provided by the reviewers, whom we sincerely thank for their valuable input in improving the quality of the manuscript.**

**All modifications made to the documents have been highlighted (in yellow).**

**First, we present the specific comments that were made as annotations in the PDF file. The authors' responses (in blue) are placed below each comment.**

**L28 – 30:** re-structure the sentence: "The model, including production functions (growth and reproduction), damage functions (diseases) and control of damage functions (treatments and vaccination), reproduced herd dynamics on a week-animal basis over a 10-year period."

**Authors**: The sentence has been restructured

**L35-36:** correct this sentence fragment. the use of "respectively" at the end of the sentence should correspond to the two prevalence rates, which need to be specified.

**Authors**: The sentence has been corrected and rephrased

L69-72 : In its current form, the research gaps are not explicit. What makes your study different from these studies. Highlight these differences as a research gap.

**Authors**: Two paragraphs have been added to contextualize the bioeconomic models used for economic questions related to beef farm management (L89-102).

L288 : Can the authors show the trend (plot) of herd dynamics at each time step till the 520 weeks?

**Authors**: A graph has been added in the appendix (S1 Figure 1). It represents the indicator of animal presence in the farm, averaged per year and per scenario.

At this stage, results per week cannot be presented. Each scenario is simulated 100 times over 520 weeks, and only the mean and standard deviation (per year, across 100 repetitions) are exported in the simulator’s result file.

L304: which time frame is referred to as interim period?

**Authors**: We refer to the gap between the age at which animals reach their selling weight and their actual selling age.

L316 – 317 : a graphical representation of this reduced prevalences would be a good addition to the result section

**Authors**: A graphical representation of the reduction in the prevalence of respiratory diseases and diarrhea has been added in the appendix (S1 Figure 2)

L387 : a graphical representation of the results will give a more comprehensive picture.

**Authors**: A graphical representation of the reduction in calf’s mortality rates has been added in the appendix (S1 Figure 3)

L393 : Are morbidity losses modelled as part of the weight gain dynamics?

The weight losses from infected animals as they transition to a recovery state will help capture the carcass value loss dynamics. I will encourage the authors to consider incorporating morbidity losses to the model.

**Authors**: Morbidity losses are accounted for in the model through a reduction in average daily gain (ADG) over a specific period (Table 4). Carcass value losses refer to the monetary value of the carcass and its conformity with slaughterhouse standards.

Lines 403–407: The term has been clarified and the paragraph has been reformulated.

**Second, reviewers’ general comments are addressed bellow.**

**Reviewer #1:**

This manuscript attempts to measure the economics of vaccinating beef animals in France. The basic simulation model is fairly standard and appears to be done correctly. However, the authors oversell the economic benefits of vaccinating calves and cows by focusing on 1 economic measure, return on investment (ROI). Using this measure alone appears to make the vaccinating of these animals as "feasible" using ROI, but ROI is not the only measure farmers will use to make decisions. When broken down the different vaccination scenarios have marginal impacts on revenue and or cost, ranging from <1% to < 4% for revenue and from 2.5% to 4% increases in costs, leading to between 1 and 4% increases in net revenue. Thus, one must ask do these numbers make the proposed vaccination "really" profitable when one factors in time and labour for farmers to undertake the vaccination programs presented, particularly in the low risk scenarios.

**Authors:** Thank you for this remark. Indeed, it is important to emphasize that decision-making in livestock farming is not based solely on financial criteria. A purely financial interpretation is insufficient to support decision-making.

We felt it was necessary to calculate this indicator, as it serves as an important argument in the veterinarian–farmer dialogue. Its formulation clearly conveys to the farmer the potential gain if they choose to adopt the preventive strategy proposed by the veterinarian.

Moreover, it was mentioned once in the Results section to present the outcomes and highlight a negative effect in a low-risk situation (L 348).

It was also referenced once in the Discussion to place our findings in context with the only economic analysis of vaccination conducted in France on the same type of production system (Discussion, L425–426 – Arnoux et al. (2021)).

Other economic outcomes were presented beyond this financial impact indicator, particularly in terms of labor productivity and gains associated with reduced antimicrobial use (AMU). Non-financial results are also discussed.

To guide the reader toward a multi-criteria analysis (economic, AMU, and farmer workload), a discussion of how our model compares with the existing literature was added in the Introduction (Lines 89–102).

A minor comment/question is Prod_revenue in equation 4, the same as Total_Revenue in equation 2? If not explain why? If so then it needs to be changed in either equation for consistnecy.

**Authors:** The term Prod_revenue has been replaced by Total revenue in Equation 4.

**Reviewer #2**

This is an interesting and insightful paper. While the paper is well-written. and comprehensive, there are key areas that the authors ought to consider to improve the robustness of their analysis and the theoretical contributions of the paper.

First, authors should highlight what differentiate the paper (esp. the model) from other research that looked at the same topic. By doing so, the research gap will be explicitly highlighted to show the theoretical contribution of the paper.

**Authors:** The developed model has been positioned in relation to existing models designed for health management issues in beef farms. The contribution of our holistic modelling approach lies in its ability to support multi-criteria evaluation and the assessment of trade-offs between different diseases management strategies simultaneously.

Second, authors may have to consider differentiating the herd based on the health status - susceptible, infected, recovered, and vaccinated. Such segmentation will allow authors to highlight the morbidity (carcass value) losses, which is currently not captured in. the model.
**Authors:** We agree on the relevance of an SIR/SIRV-type epidemiological modelling approach for animal diseases. Our bioeconomic model incorporates this simulation logic, adapted to a set of multifactorial production diseases (simulated simultaneously) for all agents and with a weekly simulation time step. This infection dynamic is described in the disease function (Diseases: Risks and Impacts: L182–L215). The description of the vaccination function is included in the treatment functions section (Treatment Functions: L217–246).

We regret that the term "carcass value loss" may be confusing with morbidity losses. We do indeed incorporate direct morbidity losses as a reduction in ADG over a given period. These disease-related losses are indicated in Table 4, column ΔADG (duration). The sentence in the discussion has been reworded to specify that we are referring to an effect on conformation and downgrading of carcasses.

Lastly, graphical representation of the results will provide more insights into the disease dynamics.

**Authors:** The graphical representation of livestock dynamics and the reduction in prevalence and mortality associated with the scenarios has been added (S1 Figure 1, S1 Figure 2, and S1 Figure 3).
