# Supplementary material for: Economics of vaccination against diarrhoea and respiratory diseases in French cow calves’ systems: A modelling approach
Source: PLoS One. 2025 Aug 6;20(8):e0329325. doi: 10.1371/journal.pone.0329325 (PMC12327687; doi:10.1371/journal.pone.0329325)
Supplement: Attachment — Submitted filename: Response to reviewers 2025.07.11.docx [file pone.0329325.s005.docx]

**Reviewer #1:**

The revisions to the manuscript are acceptable, however the format of tables needs a lot of work. Tables are required to stand alone without the reader having to refer to the text to understand the content therein. The authors use variable names that are incoherent without referring to the text, and many columns have no heading, thus the reader is left to try and work out what the numbers in the table are, whether they are monetary values or percentages. These need to be corrected before the manuscript is suitable for publication

**Authors :**

Thank you for this comment. We have revised the two calibration tables for diseases and vaccination scenarios, as well as the two result tables. We believe that Tables 1 to 3 are now clear and comprehensive.

Table 4: Some labels have been reformulated and explanatory footnotes have been added or clarified.
Table 5: An explanatory footnote on vaccine effects has been included.
Tables 6 and 7: The missing header row has been added. For several indicators, both the simulation result (unit of the indicator) and its variation relative to the reference scenario are now provided. This clarification should address the confusion between absolute and percentage values.

**Reviewer #2:**

The authors have adequately addressed the previously raised concerns. However, the manuscript lacks figures to confirm whether the graphical trends of certain results align with the reported findings. I recommend including appropriate figures to illustrate these trends for clarity and completeness.

Aside from this, I am satisfied with the current version of the manuscript.

**Authors :**

Thank you for your comment.

For this manuscript, we opted for a primarily tabular representation to facilitate the simultaneous reading of various zootechnical, epidemiological, and economic indicators.

We agree that the paper lacks graphical representations. A figure showing the average herd size was included for the validation of simulated herd dynamics (S1 Figure 1). In response to your comment, we have now added figures illustrating the simulated annual number of diarrhea and respiratory disease cases, with and without vaccination (Dia and BRD) respectively (S1 Figure 4).

If your comment refers to the trends for each individual simulation within a given scenario, unfortunately we are unable to display them without re-running the model (which would require adjustments to the code), as we only export the annual means and standard deviations for each scenario (shown in S1 Table 1 for the general indicators).
